# A Lagrangian cylindrical coordinate system for characterizing dynamic surface geometry of tubular anatomic structures

**DOI:** 10.1007/s11517-018-1801-8

**Published:** 2018-03-03

**Authors:** Torbjörn Lundh, Ga-Young Suh, Phillip DiGiacomo, Christopher Cheng

**Affiliations:** 1 Division of Vascular Surgery, Stanford University, Stanford, CA USA; 2 Department of Mathematical Sciences, Chalmers University of Technology and University of Gothenburg, 412 96 Gothenburg, Sweden; 3 Department of Bioengineering, Stanford University, Stanford, CA USA

**Keywords:** Vascular system, Cylindrical coordinates, Lagrangian, Surface curvature, Eccentricity

## Abstract

Vascular morphology characterization is useful for disease diagnosis, risk stratification, treatment planning, and prediction of treatment durability. To quantify the dynamic surface geometry of tubular-shaped anatomic structures, we propose a simple, rigorous Lagrangian cylindrical coordinate system to monitor well-defined surface points. Specifically, the proposed system enables quantification of surface curvature and cross-sectional eccentricity. Using idealized software phantom examples, we validate the method’s ability to accurately quantify longitudinal and circumferential surface curvature, as well as eccentricity and orientation of eccentricity. We then apply the method to several medical imaging data sets of human vascular structures to exemplify the utility of this coordinate system for analyzing morphology and dynamic geometric changes in blood vessels throughout the body.

**Graphical abstract:**
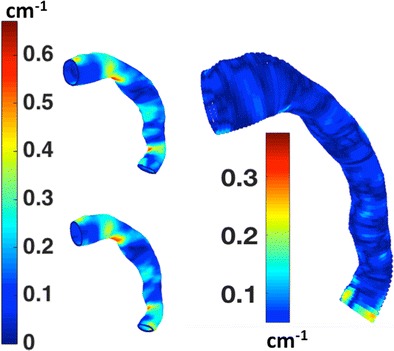
Pointwise longitudinal curvature of a thoracic aortic endograft surface for systole and diastole, with their absolute difference.

Pointwise longitudinal curvature of a thoracic aortic endograft surface for systole and diastole, with their absolute difference.

## Introduction

Accurate description of vascular geometry is important for understanding vascular anatomy, physiology, and pathology. Morphologic changes to vascular anatomy are commonly associated with clinical diagnosis and assessment. In the case of aortic aneurysms, for example, the simple measurement of aneurysm diameter can be a predictor of rupture, and aneurysm volume has been shown to correlate with the risk of clinical sequelae [3, 14]. Moreover, descriptions of dynamic anatomy, in the form of vascular deformations due to cardiac, respiratory, and musculoskeletal influences, can provide further insight into the physiological and pathological processes associated with disease development. For example, radial aortic compliance can help characterize degenerative disease in the aorta and lower extremities [4, 5]. Axial deformation and elasticity of the superficial femoral artery can be used as an indicator of lower extremity arterial health and a predictor of stent fracture [6]. Also, degree of in vivo deformation of implanted stents provides insight to predict long-term performance of the stents inside stenotic arteries [20].

Three-dimensional (3D) medical imaging is capable of providing exquisite geometric information, from which 3D geometric models can be constructed. These geometric models can then be used to quantify vascular deformation for device evaluation and development as well as perform hemodynamic and vessel structure simulations [2, 6, 8–10, 12, 13, 21–24]. One of the most established 3D lumen modeling methods is based on centerline construction, orthogonal 2D segmentations, and surface lofting [28]. While these methods allow for analysis of motion and deformation of lumen centerlines and cross sections, they lack the ability to robustly and fully characterize 3D vascular surface geometry. For example, they cannot fully quantify the variation in surface curvature along a highly curved vessel, such as in the aortic arch.

Developing more nuanced methods to quantify 3D geometric and morphological features of the human vascular system, and their dynamic changes, is needed to better understand how devices interact with the vascular system and the biomechanical characteristics that determine a patient’s prognosis and potential response to treatment. For example, recent efforts in surface modeling and analysis have demonstrated excellent promise for better predicting aortic aneurysm rupture [12, 16, 18, 19]. However, because the key parameter for evaluating mechanical fatigue of a medical device is based on alternating strain at a particular material points, a Lagrangian-based method to quantify deformation is also warranted. Here we present a method for creating a Lagrangian description of approximate cylindrical structures based on a cylindrical coordinate system. Building from a vessel centerline and lumen cross-sectional contours, this coordinate system can describe complex surface geometry, including longitudinal and circumferential curvature, cross-sectional eccentricity, and the orientation of eccentricity. We validate this method with idealized software phantoms and demonstrate the wide potential of this method by analyzing dynamic changes of blood vessel geometry in patient-specific examples of the thoracic aorta, abdominal aorta, and iliofemoral vein.

## Methods

### Formulation

**Fig. 1:**
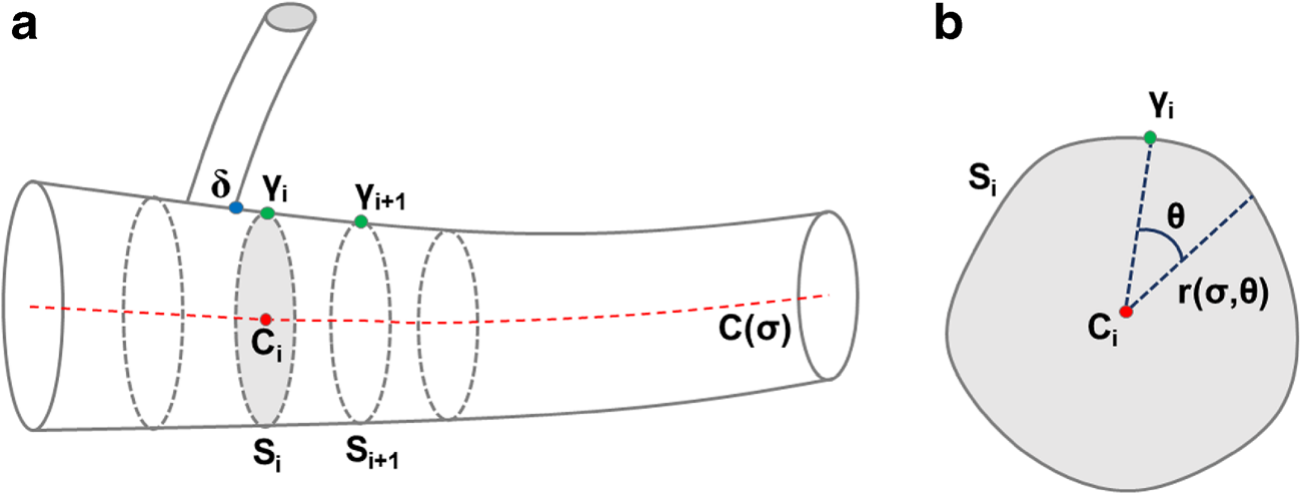
Definition of **a** contours S_i_, contour centroids *C*_i_, and Greenwich points *γ*_i_ along the longitudinal axis of the tubular structure and **b** the Greenwich point *γ*_i_ on each section *S*_i_ from which the angular dimension *θ* can be defined

To develop a Lagrangian cylindrical coordinate system which can accurately quantify the surface of a tubular anatomical structure, the first step is to develop a continuous coordinate system for the longitudinal and angular dimensions. The luminal surface of a tubular structure, such as a blood vessel, can be defined by a sequence of *n* cross-sectional contours *S*_*i*_, in 3D Cartesian space (Fig. 1a). These contours, formally denoted as ${\left\{{S}_i\right\}}_{i=1}^n$, are defined as ${S}_i={\left\{{b}_{ij}\right\}}_{j=1}^{m_i}$ where *b*_*ij*_ = (*x*_*ij*_, *y*_*ij*_, *z*_*ij*_) defines the *m*_*i*_ individual points on each contour. For each contour, the centroid ${C}_i=\left({\overline{x}}_i,\kern0.5em {\overline{y}}_i,\kern0.5em {\overline{z}}_i\ \right)$ can then be used to construct a centerline curve ${\left\{{C}_i\right\}}_{i=1}^n$. The arc length of this centerline curve, *σ*, is used as the longitudinal coordinate for this coordinate system.

**Fig. 2:**
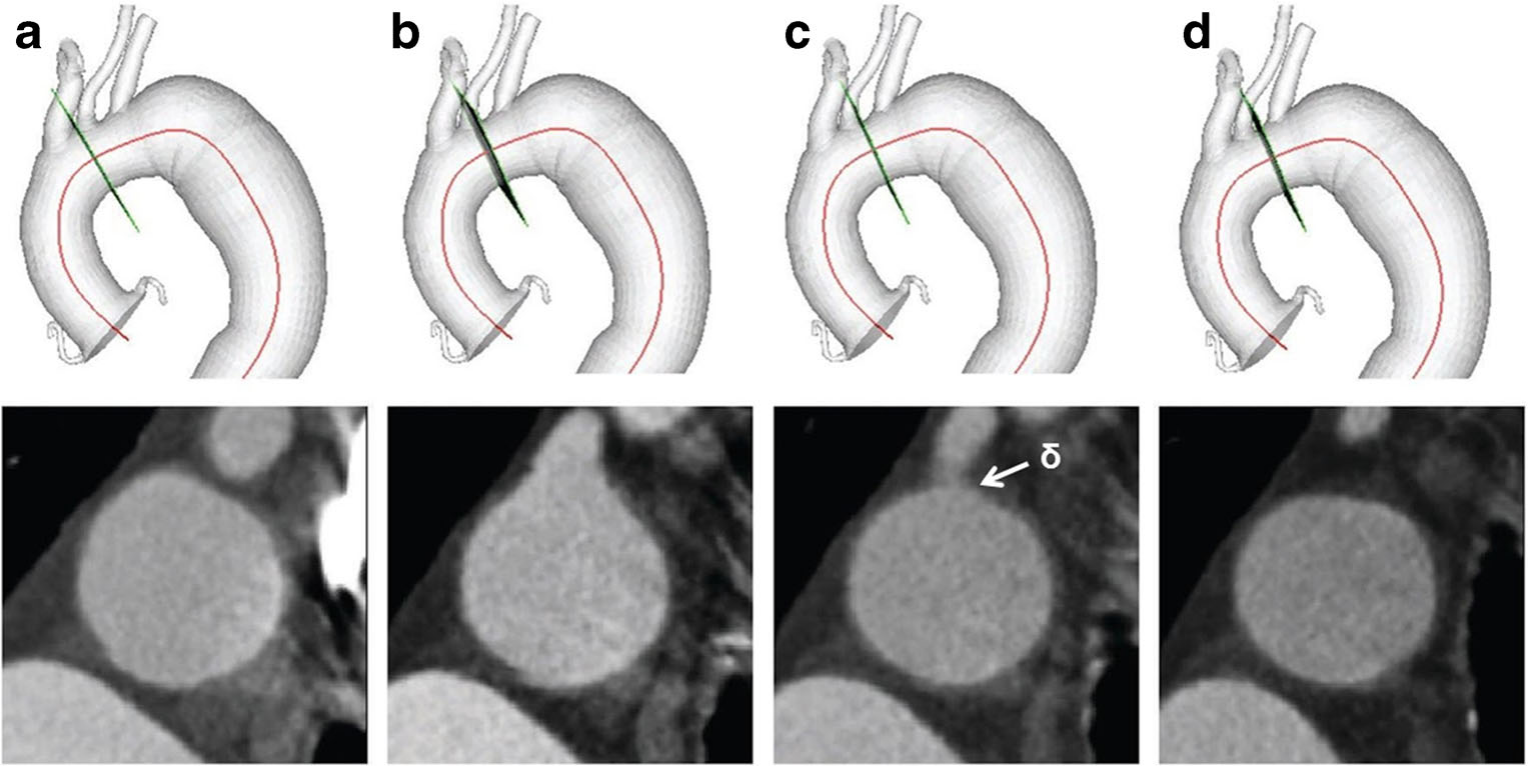
Location of the Greenwich point *δ* on a CT-based anatomic model. Orthogonal cross-sections to the mother vessel centerline **a** completely proximal to the daughter vessel where the daughter vessel is not visible, **b** at the daughter vessel where the daughter vessel appears as a “bud,” **c** at the most distal intersection of the mother and daughter vessels where the intersection point is chosen as *δ*, and **d** completely distal to the daughter vessel where the mother and daughter vessels are separate

Next, the angular coordinate, *θ*, can be defined by specifying a particular circumferential reference point among the boundary points *b*_*ij*_ for each contour *S*_*i*_, which is called *γ*_*i*_ (Fig. 1b). To define this reference point *γ*_*i*_ in a consistent, non-arbitrary way, an initial material point must be identified with a fiducial marker. For vascular structures, a bifurcation point serves as a natural fiducial marker. The lumen boundaries of the mother and daughter branches are defined as two separate sets and the most distal intersection of these two sets is selected as the material point *δ* and will be the landmark on the mother vessel that will serve as the natural reference point (Fig. 2). Then, the closest boundary point to this landmark is denoted the Greenwich point *γ* of the vessel. That is, *γ* ∈ {*b*_*ij*_} where ∣*δ* − *γ* ∣  = min_*i*, *j*_ ∣ *δ* − *b*_*ij*_∣. Now, suppose that *γ* is selected on the *k*th contour *S*_*k*_. This point is used as the reference point for the centerline curve, such that *C*(0) = *C*_*k*_ and *C*(*σ*) is proximal to *S*_*k*_ if *σ* < 0 and distal to *S*_*k*_ if *σ* > 0. Corresponding reference points on every contour can then be assigned by letting *γ*_*k*_ = *γ*, and defining *γ*_*k* + 1_ using a projection onto the *S*_*k* + 1_ section as explained in more detail later. Iterating this process defines Greenwich points for each contour. The piecewise linear curve defined by these points is defined as the Greenwich curve.

Using the longitudinal *σ* and angular *θ* dimensions, the Lagrangian cylindrical surface function *r*(*σ*, *θ*) is created (Fig. 1b). By using linear interpolation in both dimensions, a continuous vessel coordinate system provides a Lagrangian description of every vessel boundary point. The radial function *r*(*σ*, *θ*) can be expanded to include a time parameter, i.e., *r*(*σ*, *θ*, *t*), in order to define changes of the vessel surface with respect to time.

**Fig. 3:**
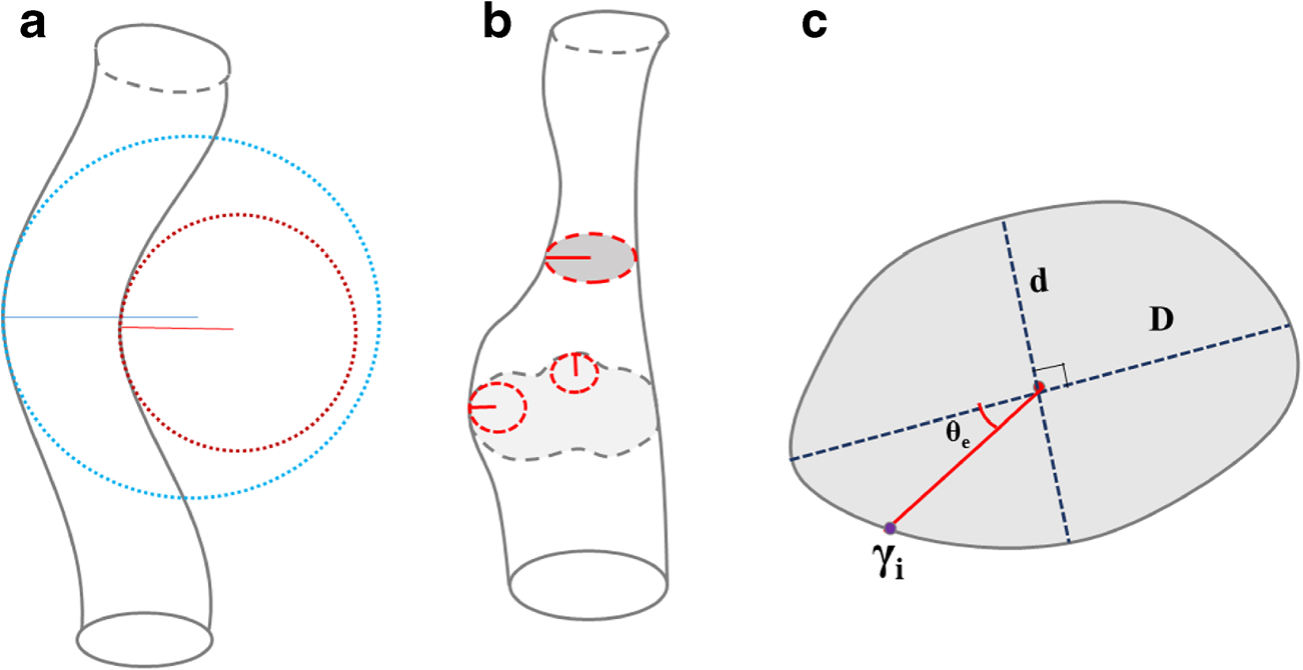
Definition of **a** longitudinal curvature (outer curve in blue and inner curve in red), **b** circumferential curvature, and **c** eccentricity, $\sqrt{1-{d}^2/{D}^2}$, and orientation of eccentricity (*θ*_e_) with respect to the Greenwich point *γ*_i_, where *d*/*D* is minimized to find the major and minor axes (color figure online)

This Lagrangian coordinate system can be used to quantify and monitor the surface curvature in both the circumferential (*θ*) and longitudinal (*σ*) directions. To compute the curvature at a given surface point, a circle is fit around the initial point and two of its symmetric neighboring points in the direction of interest (Fig. 3a, b). The reciprocal of the radius of this circle is defined as the magnitude of the curvature. The product and mean of the circumferential and longitudinal curvatures at a given point could be used to serve as proxies for the Gaussian curvature and mean curvature, respectively.

Additionally, this system can be used to quantify both the orientation and magnitude of the eccentricity of the structure. Classically, the eccentricity of an ellipse with major axis *a* and minor axis *b*, defined by $\frac{x^2}{a^2}+\frac{x^2}{a^2}=1$, has eccentricity $\sqrt{1-\frac{b^2}{a^2}}$. This definition can be generalized to irregular contours by selecting the two orthogonal diameters through the center point *C*_*i*_ while minimizing the quotient $\frac{d}{D}$ where *D* is the major axis and *d* is the axis perpendicular to *D* and defining the eccentricity as $\sqrt{1-\frac{d^2}{D^2}}$. The direction of eccentricity is defined as the positive angle *θ*_*e*_ between the major diameter *D* and the Greenwich point *γ*_*i*_ (Fig. 3c). By tracking the direction of eccentricity as a function of *σ*, the static and dynamic spirality of eccentricity can be quantified.

### Optimization of methods

**Fig. 4:**
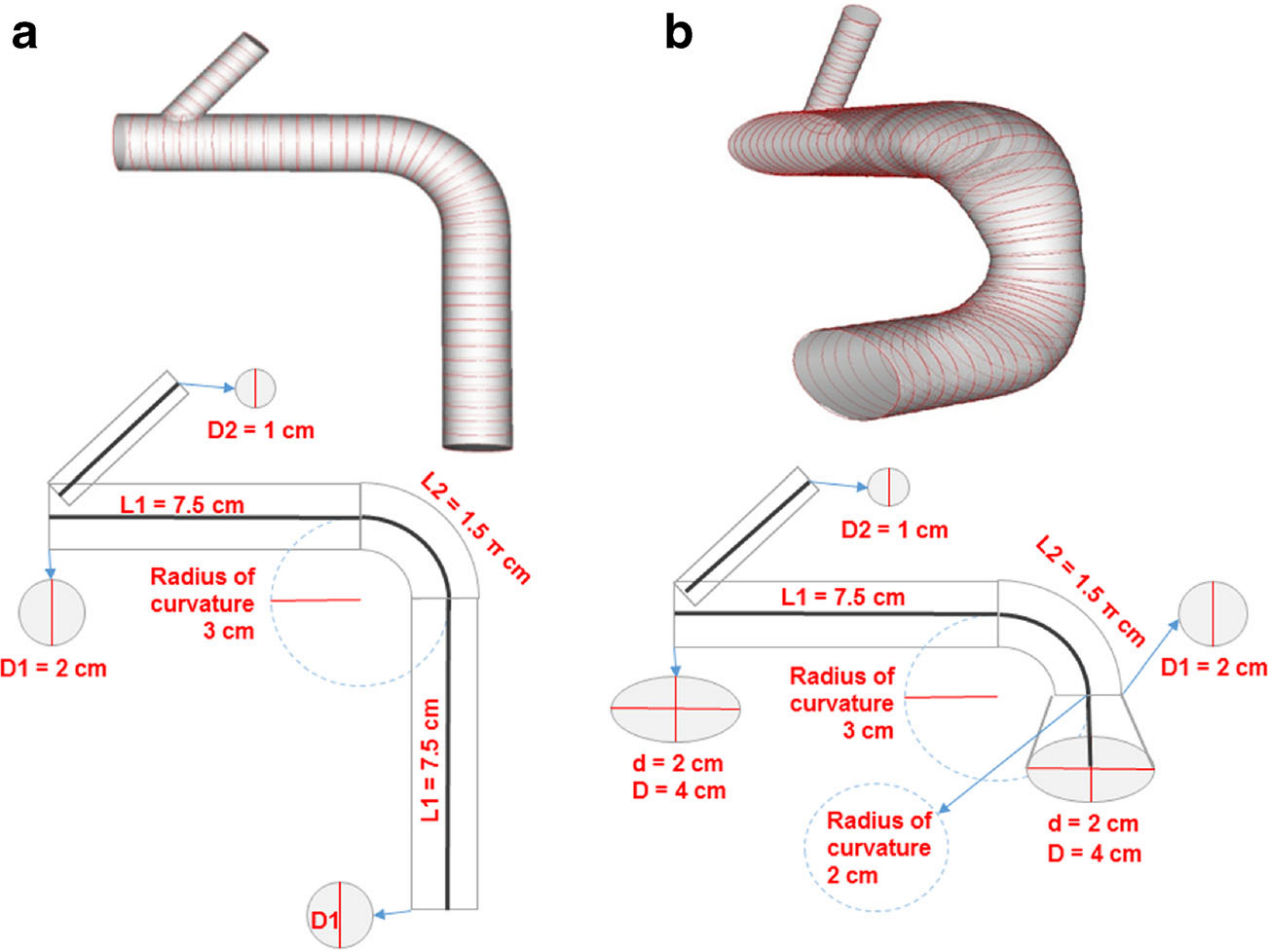
3D models (top) and schematics (bottom) of idealized software phantoms of **a** a simple phantom with a 90° bend and uniform circular cross sections and **b** a complex phantom with two 90° bends in different planes and varying non-circular cross sections

In order to apply this method to arbitrary complex tubular structures, derivation of the Greenwich curve and prescribed window sizes to compute curvature need to be optimized. For this optimization exercise, two idealized software phantoms were utilized. The first was a simple tubular phantom with circular contours and a longitudinal bend causing variation in longitudinal curvature (Fig. 4a). The second phantom was designed with non-circular cross sections and two bends in different planes, exhibiting variable circumferential curvature, longitudinal curvature, eccentricity, and orientation of eccentricity (Fig. 4b).

For defining the points that comprise the Greenwich curve, three methods were attempted. The first method started with the initial point γ_k_ and projected the vector *γ*_*k*_ − *C*_*k*_ onto the *S*_*k* + 1_th section along the vector *C*_*k* + 1_ − *C*_*k*_. Then, γ_*k* + 1_ is selected to be the closest point in the sequence ${\left\{{b}_{\left(k+1\right)\ j}\right\}}_{j=1}^{m_{k+1}}.$ The second method instead projected the vector *γ*_*k*_ − *C*_*k*_ along the normal of the *S*_*k*_th section onto the *S*_*k* + 1_th section and defined *γ*_*k* + 1_ in the same way. The third method simply selected *γ*_*k* + 1_ to be the closest point in ${\left\{{b}_{\left(k+1\right)\ j}\right\}}_{j=1}^{m_{k+1}}$ from γ_k_. Using any of these methods, Greenwich points on every section can be defined. The piecewise linear curve formed by these Greenwich points is then designated as the Greenwich curve. In this paper, we selected the first method, i.e., projection of the vector *γ*_*k*_ − *C*_*k*_ onto the *S*_*k* + 1_th section along the vector *C*_*k* + 1_ − *C*_*k*_, since we would like to obtain a Greenwich curve that more robustly tracks the centerline curve.

To determine the optimal span length of the three points to calculate curvature (i.e., window size), the idealized phantoms, where the analytic curvatures were known, were used. A variety of window sizes were evaluated for both the longitudinal and circumferential directions and window sizes which adequately resolved the true curvature, yet did not produce substantial spurious oscillations, were selected.

### Application to phantoms and human data sets

**Fig. 5:**
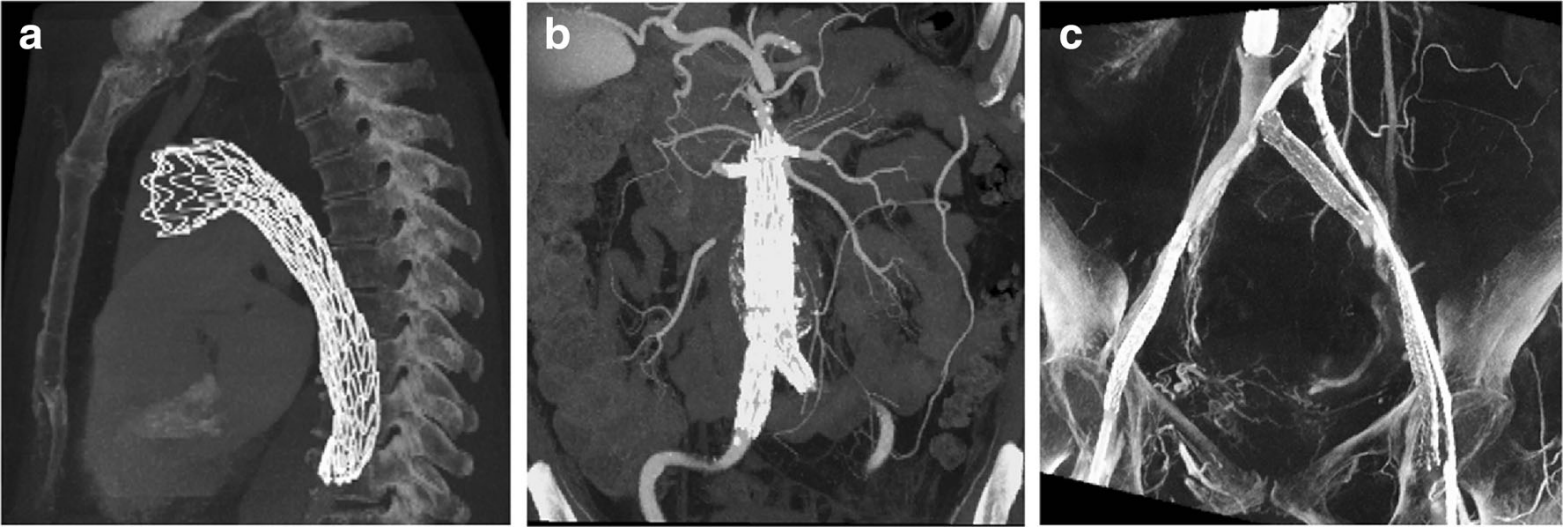
Human data sets: 3D high-resolution computed tomography data of a **a** thoracic aorta, **b** abdominal aorta, and **c** iliofemoral veins after endograft implantation

The optimized coordinate system was applied to the two phantoms described above, as well as three human data sets to exemplify the full range of quantifications and analyses possible. For the human data, high-resolution computed tomography (CT) imaging data were acquired of a thoracic aortic endograft implanted via thoracic endovascular aortic repair (TEVAR) (Fig. 5a), an abdominal aorta and the visceral artery branches after endovascular aneurysm repair (EVAR) (Fig. 5b), and the iliofemoral veins after stent implantation (Fig. 5c). From the idealized phantoms and the human data sets, centerlines and orthogonal segmentations were created using SimVascular (Open Source Medical Software Corporation, San Diego, CA) [28]. Modeling was started by creating an initial lumen path manually along the center of vessel lumens. Then, semi-automatic 2D level set segmentation was performed orthogonally to this initial path, at every one half radius of the vessel. From every segmentation contour, mathematical centroid was extracted and connected to form the centerline. To assure the orthogonality of lumen contours, subsequent round of 2D segmentation was performed along the centerline instead of initial path, with consistent interval. The centerline was updated according to the second-round contours, which together used as the input for this application. The cylindrical coordinate system methods were applied to quantify the longitudinal and circumferential curvature, as well as the amplitude and direction of eccentricity at each cross section. Finally, for the thoracic aortic endograft, dynamic changes in geometry due to cardiac pulsation are shown by comparing the surface geometries between systole and diastole.

## Results

### Optimization of parameters

**Fig. 6:**
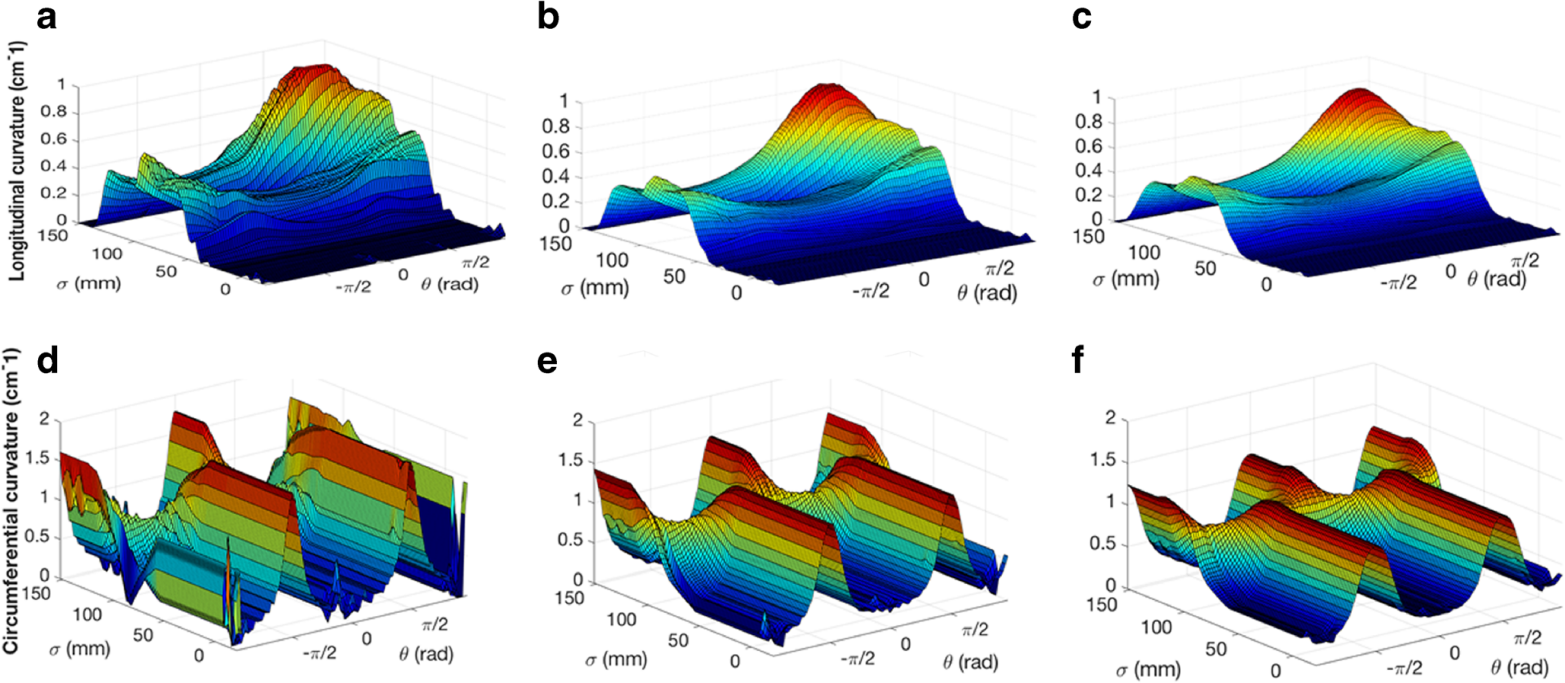
Illustration of window size optimization for calculating surface curvature of the complex idealized software phantom shown in Fig. 4b: the pointwise calculations of longitudinal curvature for three different window sizes in the sigma direction, **a** 20 mm, **b** 30 mm, and **c** 40 mm, and the pointwise calculations of circumferential curvature for three different window sizes in the theta direction, **d** π/8 rad, **e** π/4 rad, and **f** 3π/8 rad

Figure 6 illustrates a subset of the trials run to optimize window sizes to calculate the surface curvature in the longitudinal (Fig. 6a–c) and circumferential (Fig. 6d–f) directions. For longitudinal curvature, a window size of 20 mm produced spurious noise, as indicated by the deep ridges in Fig. 6a, while a window size of 40 mm underestimated the peak longitudinal curvature of 1 cm^−1^ on the inner curve of the second bend by 7% (Fig. 6c). A longitudinal window size of 30 mm, however, did not exhibit spurious noise and was able to calculate the peak curvature to within 0.5% (Fig. 6b). In the circumferential direction, a window size of π/8 exhibited spurious oscillations (Fig. 6d) while a window size of 3π/8 underestimated the peak circumferential curvature of 2 cm^−1^ on the elliptical cross sections by 35% (Fig. 6f). In this case, a window size of π/4 rad (Fig. 6e) was determined to be optimal. Similar relative window sizes were utilized for analysis of idealized phantom and human data sets.

### Application to idealized software phantoms

**Fig. 7:**
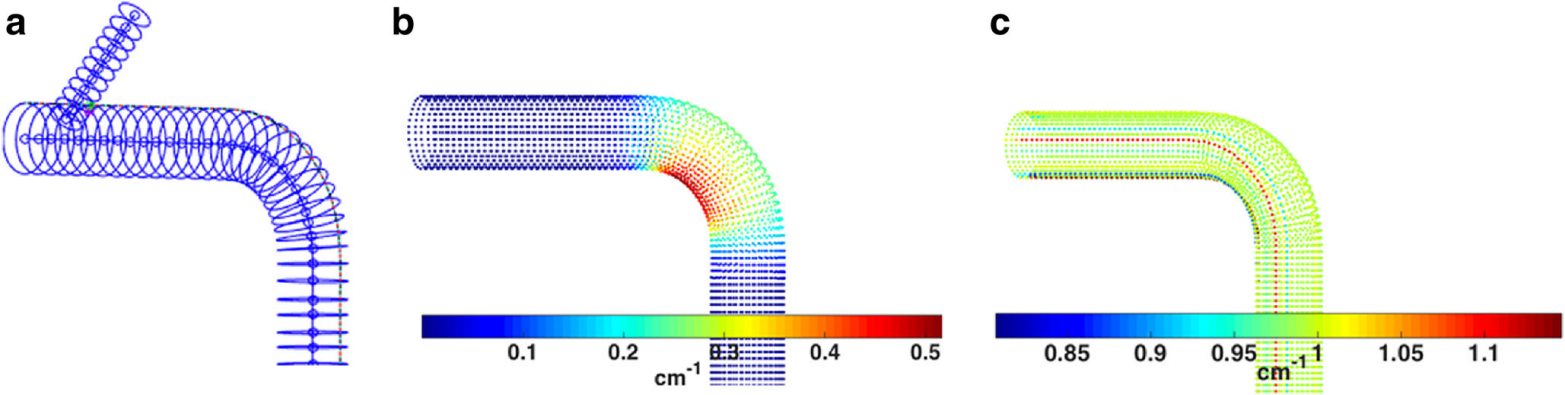
Results of the analyses for the simple idealized phantom from Fig. 4a, including **a** 2D cross-sectional contours and centerlines of the main vessel and branch (blue) and Greenwich curve (thin black line), **b** longitudinal curvature mapped pointwise onto the surface of the 3D structure, and **c** circumferential curvature mapped pointwise onto the surface of the 3D structure (the window sizes for the curvature computations are 30 mm for longitudinal and π/4 rad for circumferential) (color figure online)

The constructed 3D model of the simple phantom with 2D cross-sectional contours, centerlines, and Greenwich curve is shown in Fig. 7a. The pointwise measurements of the longitudinal and circumferential curvature along the entire surface are shown in Fig. 7b, c. As shown in Fig. 7b, the longitudinal curvature was calculated to be 0 cm^−1^ proximal and distal to the bend and a maximum value of approximately 0.5 and 0.25 cm^−1^ along the inner and outer curve of the bend, respectively. In Fig. 7c, the circumferential curvature was calculated to be approximately 1.0 cm^−1^ along the entire surface.

**Fig. 8:**
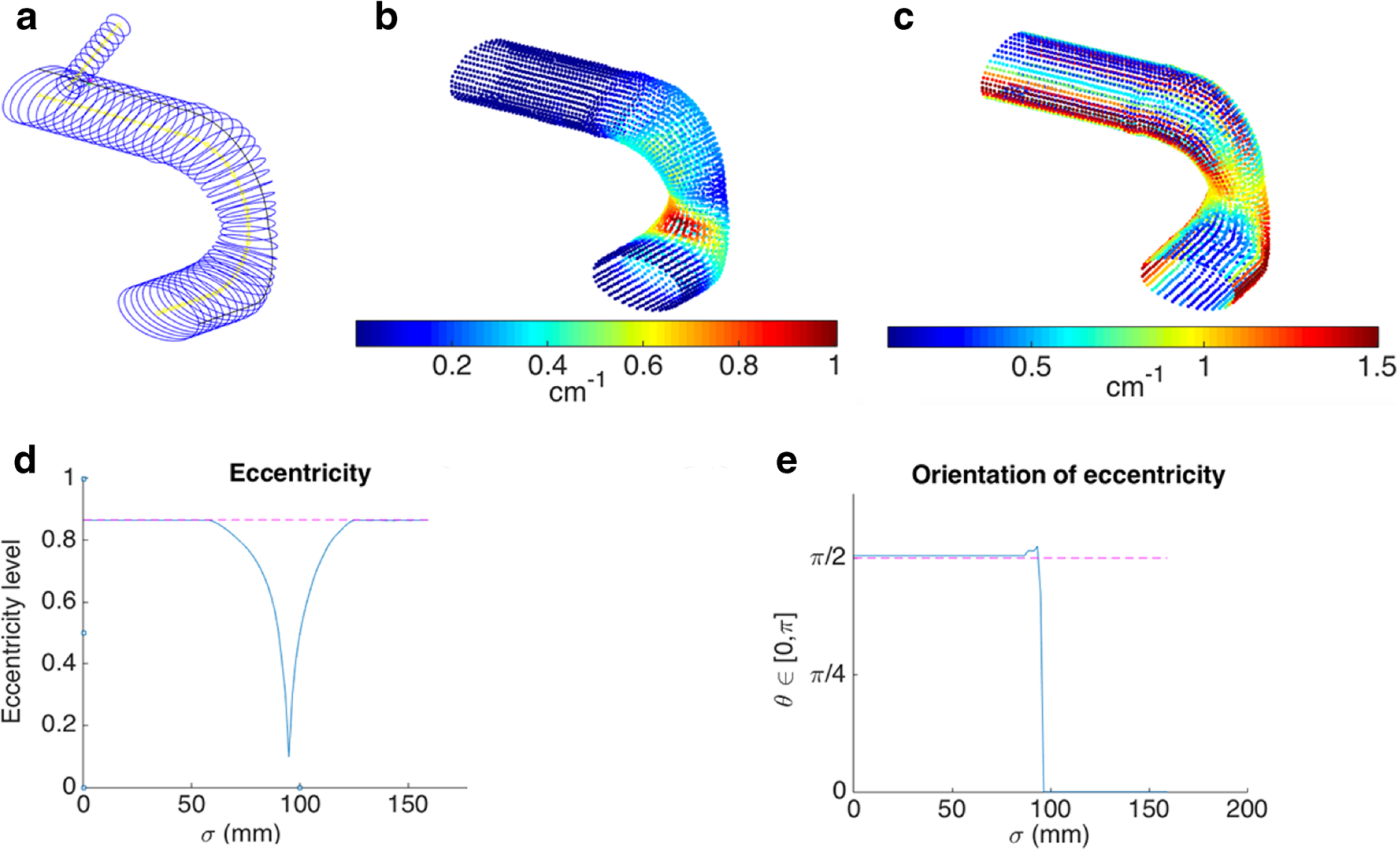
Results of the analyses for the complex idealized phantom from Fig. 4b, including **a** 2D cross-sectional contours (blue) and centerlines (yellow) of the main vessel and branch and Greenwich curve (thin black line), **b** longitudinal curvature mapped pointwise onto the surface of the 3D structure, **c** circumferential curvature mapped pointwise onto the surface of the 3D structure, **d** magnitude of eccentricity along the centerline arc length, and **e** orientation of eccentricity along the centerline arc length (the window sizes for the curvature computations are 30 mm for longitudinal and π/4 rad for circumferential) (color figure online)

Likewise, the constructed model of the complex phantom is shown in Fig. 8a and the results of the analyses are shown in the remainder of Fig. 8. Surface calculations of longitudinal and circumferential curvature are shown in Fig. 8b, c, and the quantification of magnitude and orientation of eccentricity are shown in Fig. 8d, e. As shown in Fig. 8b, the straight sections exhibit 0.0 cm^−1^ longitudinal curvature, the outer curve of the first bend exhibits 0.5 cm^−1^ curvature, and the inner curve of the second bend exhibits 1.0 cm^−1^ curvature. Figure 8c depicts circumferential curvatures of 1.5 cm^−1^ along the vertices of the major of the elliptical cross sections, approximately 0.25 cm^−1^ along the flat sections of the elliptical cross sections and 1.0 cm^−1^ at the circular cross sections. In Fig. 8d, the magnitude of eccentricity along the longitudinal direction was calculated as 0.87 for the proximal and distal straight sections, with a steep dip at *σ* = 96 mm, reflecting the location of the circular cross sections. In Fig. 8e, the orientation of eccentricity was calculated as 1.6 and 0.0 rad for the proximal and distal straight sections, respectively, with a sudden transition at *σ* = 96 mm. As complement to these images, Table 1 shows the absolute difference between the numerically estimated curvatures and the analytic solution with respect to minimum, maximum and median.

**Table 1:** Absolute curvature difference between numerical estimation and analytic solution

Absolute curvature difference (cm^−1^)	Minimum	Maximum	Median
Simple phantom, longitudinal curvature	5.7 × 10^−8^	0.53	8.8 × 10^−4^
Simple phantom, circumferential curvature	1.9 × 10^−4^	0.24	0.0094
Complex phantom, longitudinal curvature	1.9 × 10^−6^	1.0	0.13
Complex phantom, circumferential curvature	0.00091	1.7	0.47

### Application to human image data sets

**Fig. 9:**
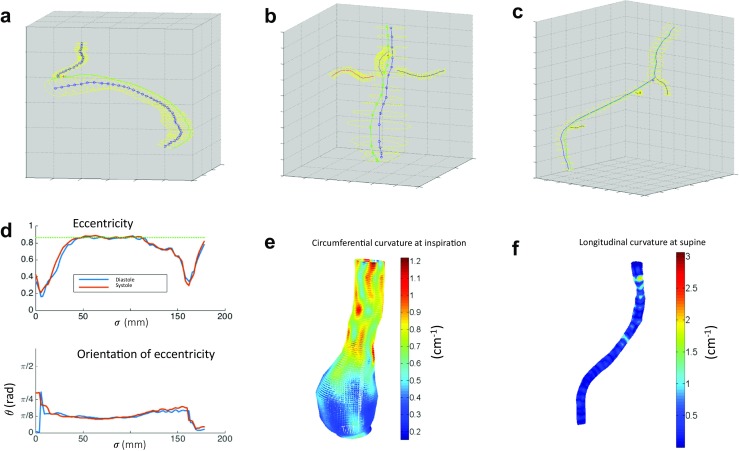
Results of the analyses for the human CT image data from Fig. 5, including 3D representations of the **a** thoracic aortic endograft during diastole, **b** aneurysmal abdominal aorta during inspiration, and **c** right iliofemoral vein in the supine position, the corresponding **d** magnitude (above) and orientation (below) of the eccentricity along the centerline of the thoracic aortic endograft, **e** circumferential curvature of the abdominal aorta, and **f** longitudinal curvature of the right iliofemoral vein

Figure 9a–c illustrates orthogonal segmentations, centerlines, and derived Greenwich curves for each human image data set. Utilizing the coordinate system method on these examples, the amplitude and orientation of the vessel eccentricity, and pointwise longitudinal and circumferential surface curvatures were quantified. Examples of these analyses can be seen in Fig. 9d–f. Figure 9d shows the amplitude and orientation of the eccentricity of the thoracic aortic endograft (from Fig. 5a). Figure 9e shows the pointwise circumferential surface curvature of the aneurysmal abdominal aorta (from Fig. 5b). Figure 9f shows the pointwise longitudinal curvature of the iliac vein (from Fig. 5c).

**Fig. 10:**
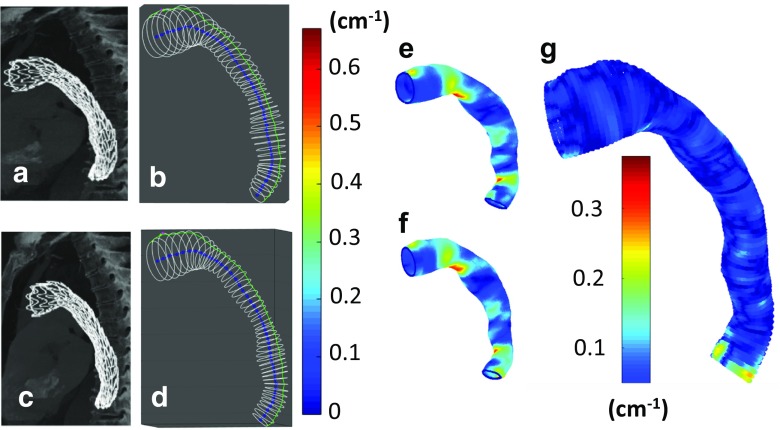
Illustration of thoracic aortic endograft in systole (top row) and diastole (bottom row) (the human CT image data from Fig. 5a). Left column shows CT images for **a** systole and **b** diastole; middle column shows 3D models of the cylindrical coordinate system for **c** systole and **d** diastole. The next column shows pointwise longitudinal curvature of the endograft surface for **e** systole and **f** diastole. Right column shows the pointwise absolute difference in longitudinal curvature between systole and diastole (**g**). Note how while it is difficult to visualize differences in longitudinal curvature by looking at the CT images or 3D models, the color maps of curvature show quantitative differences. According to the presented example of longitudinal curvature changes, cardiac pulsation induces curvature changes along the most distal aorta

To demonstrate the ability of the developed method to compare dynamic changes of surface geometry, Fig. 10 depicts a coordinate system analysis of pointwise longitudinal surface curvature for the thoracic aortic endograft (from Fig. 5a) at systolic and diastole phases of the cardiac cycle.

## Discussion

In this paper, we describe a robust method for fully describing the surface geometry of complex anatomic tubular structures by using a Lagrangian cylindrical coordinate system. The method was validated on idealized software phantoms and then applied to three human data sets of different anatomies.

To ensure a robust and widely applicable system, window sizes for curvature calculation need to be standardized based on the anatomy of interest. The window size needs to be small enough to ensure accurate estimation of maximum curvature values, yet not so small as to cause substantial spurious oscillations. Based on experiments on the complex idealized phantom in Fig. 4b, the optimal window size for longitudinal and circumferential curvature calculations was 30 mm and π/4 rad, respectively. These correspond roughly the diameter of the vessel and one eighth of the circumference, respectively. These guidelines were used for all subsequent analyses.

Application to the simple and complex idealized phantoms from Fig. 4 shows excellent agreement with analytic solutions of surface curvature and eccentricity. Figure 7 shows that the straight sections of the simple phantom were correctly calculated to have 0 cm^−1^ longitudinal curvature and that the inner and outer curvatures at the bend correctly corresponded with 2 and 4 cm radius of curvature, respectively. In addition, the circumferential curvature was correctly calculated to correspond with 1 cm radius of curvature (2 cm diameter).

Analysis of the complex idealized phantom had similar excellent correspondence with analytic solutions, such as longitudinal curvature values of 0.5 and 1.0 cm^−1^ at the outer curve of the first bend and inner curve of the second bend, respectively (Fig. 8). For the contour cross sections, the circumferential curvatures calculated for the circular cross sections and the flat sections of the elliptical cross sections were 1.0 and 0.25 cm^−1^, respectively, which perfectly match the analytic solutions. The calculated circumferential curvature of 1.5 cm^−1^ at the vertices of the ellipse is 25% below the analytic solution; however, increased point sampling around the contour would greatly improve this calculation. The analytic eccentricity of an ellipse with a 4 cm major diameter and 2 cm minor diameter is $\sqrt{3}/2$ (dotted pink line in Fig. 8d), which is almost exactly the value calculated by our method. In addition, the orientation of eccentricity calculated by the method closely approximated the π/2 and 0 rad analytic solutions (Fig. 8e). Both eccentricity graphs capture the transition point at the correct longitudinal location where the idealized model bent from one plane to an orthogonal plane.

Figure 9 shows applicability of the method to actual medical images of different vascular structures. For the thoracic aortic endograft, the eccentricity was calculated to be higher (less circular) at the ends of the graft, which makes sense due to the decrease in hoop strength at the free ends of the stent graft (Fig. 9d). In the abdominal aorta example, circumferential curvature calculations show higher curvatures at the normal proximal aorta (equivalent to diameter ≈ 2 cm), lower curvatures at the aneurysm (equivalent to diameter ≈ 6 cm), and moderate curvature at the transition between normal and aneurysmal sections (Fig. 9e). For the iliofemoral vein, the longitudinal curvature was predominantly 0.2 cm^−1^ or equivalent to approximately 5 cm radius of curvature (Fig. 9f).

The CT images and 3D geometric models of the thoracic aortic endograft in Fig. 10a–d illustrate how difficult it is to qualitatively identify subtle differences in surface geometry and morphology of vessels at different physiological states. However, with the color map of pointwise longitudinal surface curvature (Fig. 10e, f), the coordinate system analysis shows the power of its quantitative sensitivity. For example, the diastolic phase (Fig. 10f) shows a larger region of high longitudinal curvature along the inner curve of the proximal descending aorta as compared to the systolic phase (Fig. 10e). This makes sense because the during the high pressure pulse of systole, we would expect the endograft to straighten slightly.

Several methods have been developed to model human vessels and quantify geometric curvature. Classic methods have employed 2D level set segmentation creating a set of orthogonal contours following vascular lumen, acquiring centerlines from the contours, and computing the curvature along the centerline [6, 7, 21, 22, 24, 25]. Other available methods include 3D segmentation with growing seeds, acquiring centerline from inscribing spheres in lumenal surface, and computing the centerline curvature [11, 15]. It is also possible to acquire surface curvature directly across 2D surface patches such as a built-in function provided by the Vascular Modeling Toolkit (VMTK) [1]. The method presented in this study improved the classic centerline-based method by computing the surface curvature using 2D-segmented contours as an input. In addition to the traditional centerline curvature, we believe that surface curvature provides additional, relevant information for characterizing vascular dynamics and designing novel medical devices. Furthermore, the method in this study provides curvature output along surface lines, not 2D surface patches like the VMTK methods above. The main reason we computed surface curvature was to quantify inner and outer line geometries. These surface line curvatures are crucial to understand how medical device dynamically deform under in vivo condition and if in-stent restenosis occurs along the surface line with challenging geometry [29]. The methods computing surface curvature across 2D surface patches can also calculate surface line curvature with secondary calculations and averaging, but this requires additional steps and relies on averaging across 2D areas which results in lower resolution calculations.

More complete characterization of vascular geometry may help predict disease severity, such as aneurysm rupture risk [12, 16, 18, 19, 26], quantify pre- and post-operative geometric alterations to determine the mechanical impact of devices on the native anatomy [25], establish boundary conditions with which to evaluate and predict device failures due to cyclic fatigue [7], and more fully describe dynamic anatomy to come up with better device solutions. For example, in vivo arterial motions have often been implicated in cyclic mechanical fatigue and stent fracture [6, 7, 13, 17, 21]. Additional aortic endograft design challenges for aneurysm or dissection repair include cardiac-induced deformation, hemodynamic forces, and vulnerable and complex anatomies [27]. The Lagrangian coordinate system described in this paper improves our ability to evaluate deformations of anatomy and implanted devices at material points.

Because this cylindrical coordinate system method uses piecewise linear centerlines with linear interpolation between cross-sectional contours, the quality of the geometric model and analyses is dependent of the quality and quantity of the cross-sectional contours. Specifically, the coordinate system requires that the original 2D contours to be sufficiently orthogonal to the centerline and sufficiently densely-spaced. However, this method can include spline interpolation between centerline points and cross-sectional contours, relieving some of the need for densely packed contours. In addition, the method can be generalized to input a volumetric model, derived from any number of segmentation methods, and then use that model to create arbitrary cross-sectional contour densities based on need. These method extensions, along with applying these techniques to a wide range of anatomic structures including vascular, pulmonary, gastrointestinal, and reproductive, will be topics of future research.
